# Peroxisome Proliferator-Activated Receptors and Acute Lung Injury

**DOI:** 10.1155/2007/63745

**Published:** 2007-07-09

**Authors:** Rosanna Di Paola, Salvatore Cuzzocrea

**Affiliations:** ^1^Department of Clinical, Experimental Medicine and Pharmacology, School of Medicine, University of Messina, Via C. Valeria, Torre Biologica, Policlinico Universitario, 98123 Messina, Italy; ^2^Institute of Pharmacology, School of Medicine, University of Messina, Via C. Valeria, Torre Biologica, Policlinico Universitario, 98123 Messina, Italy

## Abstract

Peroxisome proliferator-activated receptors are ligand-activated transcription factors belonging to the nuclear hormone receptor superfamily. PPARs regulate several metabolic pathways by binding to sequence-specific PPAR response elements in the promoter region of target genes, including lipid biosynthesis and glucose metabolism. Recently, PPARs and their respective ligands have been implicated as regulators of cellular inflammatory and immune responses. These molecules are thought to exert anti-inflammatory effects by negatively regulating the expression of proinflammatory genes. Several studies have demonstrated that PPAR ligands possess anti-inflammatory properties and that these properties may prove helpful in the treatment of inflammatory diseases of the lung. This review will outline the anti-inflammatory effects of PPARs and PPAR ligands and discuss their potential therapeutic effects in animal models of inflammatory lung disease.

## 1. PPARs: OVERVIEW

**Figure 1:**
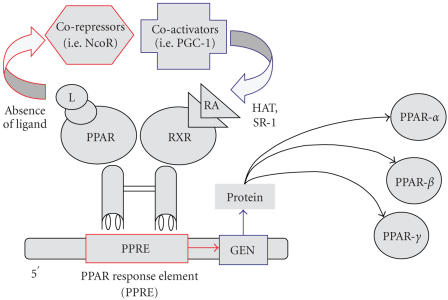
Schematic of PPAR activation events. Like other nuclear hormone receptors, PPAR acts as a ligand-activated transcription factor. PPAR-*α*, when activated after binding with specific ligand, interacts with RXR and regulates the expression of target genes. These genes are also involved in the catabolism of fatty acids. Conversely, PPAR-*γ* is activated by different ligands (e.g., prostaglandins, leukotrienes, and antidiabetic thiazolidinediones) and regulates the expression of genes involved in the storage of the fatty acids. PPAR-*β* is only weakly activated by fatty acids, prostaglandins, and leukotrienes and has no known physiologically relevant ligand. Abbreviations: nuclear corepressor protein: (NcoR); PPAR gamma coactivator 1:(PGC-1); histone acyltransferase: (HAT); steroid receptor coactivator-1: (SR-1); 9-cis retinoic acid: (RA).

PPARs are members of the nuclear hormone receptor superfamily that were initially characterized as molecules that mediated the proliferation of peroxisomes in rodent liver parenchymal cells in response to the hypolipidemic drug clofibrate [1]. Subsequently, PPARs have been shown to regulate the expression of genes involved in a variety of biological processes, including lipid metabolism and insulin sensitivity [2, 3]. Three isotypes of PPAR exist, PPAR-*α* (alpha), PPAR-*β*/*δ* (beta/delta), and PPAR-*γ* (gamma), which are encoded by three separate genes and display distinctly different tissue distributions and functions. PPAR-*γ*, like other PPAR isotypes, exists as a heterodimer complexed with the retinoid X receptor and several corepressor molecules that tonically suppress PPAR activity [4]. In the presence of PPAR ligands, corepressor molecules are shed, followed by association of coactivator proteins, binding to specific PPAR-response elements, and transcription of target genes [4] (see Figure 1).

PPAR-*α* is activated by polyunsaturated fatty acids and synthetic fibrates, and is implicated in regulation of lipid metabolism, lipoprotein synthesis and metabolism, and inflammatory response in liver and other tissues. PPAR-*α* is highly expressed in tissues with high fatty acid oxidation (such as liver, kidney, and heart muscle), where it controls a comprehensive set of genes that regulate most aspects of lipid catabolism. Like several other nuclear hormone receptors, PPAR-*α* heterodimerizes with RXR alpha to form a transcriptionally competent complex [5]. In addition, PPAR-*α* is expressed in vascular endothelial cells, smooth muscle cells, monocyte/macrophages, and T lymphocytes. Activation of PPAR-*α* in selected cellular systems increases HDL cholesterol synthesis, stimulates “reverse” cholesterol transport, and reduces triglycerides [6].

The biological role of PPAR-*β*/*δ* has not been clearly defined. Animal studies revealed that PPAR-*β*/*δ* plays an important role in the metabolic adaptation of several tissues to environmental changes. Treatment of obese animals with specific PPAR-*β*/*δ* agonists results in normalization of metabolic parameters and reduction of adiposity. PPAR-*β*/*δ* was also implicated in the regulation of fatty acid burning capacities of skeletal muscle and adipose tissue by controlling the expression of genes involved in fatty acid uptake, beta oxidation, and energy uncoupling. Moreover, PPAR-*β*/*δ* has been shown to mediate the adaptive metabolic response of skeletal muscle to endurance exercise by controlling the number of oxidative myofibers and stimulating fatty acid catabolism in muscular tissue [7]. Recent studies revealed that ligand activation of these receptors is associated with improved insulin sensitivity and elevated HDL levels thus demonstrating promising potential for targeting PPAR-*β*/*δ* in the treatment of obesity, dyslipidemias, and type 2 diabetes [8].

PPAR-*γ* plays an important role in the regulation of proliferation and differentiation of several cell types, including adipose cells. This receptor has the ability to bind a variety of small lipophilic compounds derived from both metabolism and nutrition. These ligands, in turn, direct cofactor recruitment to PPAR-*γ*, regulating the transcription of genes in a variety of complex metabolic pathways. PPAR-*γ* is highly expressed in adipocytes, where it mediates differentiation, promotes lipid storage, and, as a consequence, is thought to indirectly improve insulin sensitivity and enhance glucose disposal in adipose tissue and skeletal muscle [9, 10]. Activation by drugs of the glitazone (thiazolidinediones) group results in insulin sensitization and antidiabetic action. Naturally occurring lipids can also activate PPAR-*γ*, including arachidonic, oleic, and linoleic acid, and the cyclopentenone prostaglandin (PG) 15-deoxy Delta_12,14_-PGJ_2_ (15d-PGJ_2_), a metabolite of prostaglandin D_2_. Nitosylated oleic and linoleic acid species have more recently been identified as potent PPAR-*γ* agonists at concentrations present in human tissues. The cellular expression profile of PPAR-*γ* in pulmonary tissue has not been well characterised, but studies have uncovered abundant expression of PPAR-*γ* in airway epithelium [11], bronchial submucosa [12], in mononuclear phagocytes such as human alveolar macrophages (AM), human T lymphocytes, and in several pulmonary cell lines, including human bronchial and alveolar epithelial cells (NL20, BEAS, and A549 [13]) and human airway smooth muscle (HASM) cells [14]. The expression of the various isotypes of PPAR is highly cell specific. For instance, HASM cells express PPAR-*α* and PPAR-*γ*, but not PPAR-*β*/*δ*, whereas primary normal human bronchial epithelial cells and human lung epithelial cell lines BEAS 2B, A549, and NCI-H292 all express PPAR-*γ* and PPAR-*β*/*δ*, but not PPAR-*α* [15]. Because little is known regarding the role of PPAR-*β*/*δ* in regulating inflammation, especially in the context of lung injury, this review will focus on the biology of PPAR-*α* and PPAR-*γ* in human and animal models of acute lung injury (ALI).

## 2. ACUTE LUNG INJURY (ALI)

Injury to the lung can occur in response to a variety of pulmonary and extrapulmonary insults. In humans, ALI and its more severe form, the acute respiratory distress syndrome (ARDS) are syndromes of acute respiratory failure, which are defined clinically on the basis of both radiographical (bilateral lung field infiltrates) and physiological (the ratio of arterial oxygen pressure and the inspiratory oxygen concentration, P_*a*_/F_*i*_ ≤ 300 mmHg for ALI and ≤200 mmHg for ARDS) criteria. These syndromes occur as a result of widespread damage to cells and structures of the alveolar capillary membrane and evolve within hours to days [16]. ALI/ARDS can develop as a consequence of critical illness of diverse etiologies, including direct injury to lung such as pneumonia, aspiration, toxic inhalation, near drowning, or lung contusion; as well as indirect mechanisms, such as sepsis, burn injury, pancreatitis, gynecological insults (abruption of placenta, amniotic embolism, eclampsia), or massive blood transfusion [17].

**Figure 2:**
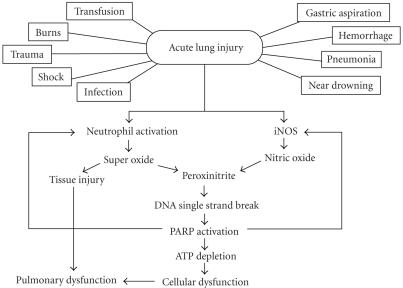
Pathophysiological events in acute lung injury.

The pathophysiological consequences of ALI/ARDS are related to the altered pulmonary capillary permeability and alveolar diffusion capacity, as well as the increased intrapulmonary shunt. Endothelial injury and increased vascular permeability is a central feature of ALI/ARDS, and some but not all studies suggest a role for neutrophils in mediating endothelial injury [17, 18]. Epithelial injury is also important not only in the development but also the repair of the ALI/ARDS [19]. The degree of epithelial injury can predict outcome of ALI/ARDS [20]. Loss of epithelial integrity and injury to type II alveolar cells can disrupt the normal fluid transport, thereby impairing the removal of fluid from the alveolar space. Injury to the type II pneumocytes can reduce the production of surfactant, which contributes to the clinical course of worsening atelectasis and gas exchange. The process of epithelial repair can be dysregulated, leading to proliferation of fibroblasts, exuberant matrix deposition and remodeling, and culminate in fibrosis [21, 22]. There are complex autocrine and paracrine interrelationships of cytokines, as well as proinflammatory mediators that initiate and amplify the inflammatory response in ALI/ARDS. The cellular responses include the expression of endothelial adhesion molecules, as well as the margination and migration of neutrophils and other inflammatory cells. A number of soluble factors are released that contribute to the pathobiology of ALI/ARDS, including cytokines, lipid mediators, proteases, oxidants, growth factors (e.g., transforming growth factors (TGFs)), nitric oxide (NO), and neuropeptides [23] (see Figure 2). This inflammatory state is driven by the activation of several key-signalling pathways including the NF-*κ*B, AP-1 and the mitogen-activated protein kinase (MAPK) pathways.

### 2.1. PPAR-*α* and lung injury

Based on both in vivo and in vitro studies in multiple cell systems, PPAR-*α* ligands have important anti-inflammatory properties. For example, treatment of an activated murine macrophage cell line with the synthetic PPAR-*α* agonist Wy14643 [peroxisome proliferation-activated receptor-alpha (PPAR-alpha) activator, 4-cholro-6-(2.3-xylidino)-2-pyrimidinaylthio acetic acid] resulted in inhibition of nitric oxide synthase (NOS), whereas LTB_4_ and 8(S)-HETE, two natural PPAR-*α* ligands, stimulated the expression of nitric oxide synthase (NOS) activity in these same cells [24]. The authors have postulated that this disparity resulted from low potency and specificity of the endogenous ligands in comparison with that of synthetic compounds [25]. The in vivo role of PPAR-*α* in the regulation of inflammatory/immune-related functions is less well studied. The first in vivo evidence for the role of PPAR-*α* evolved from studies using PPAR-*α* deficient mice [26]. These mice are viable, but exhibit altered triglyceride and cholesterol metabolism and fail to respond to appropriate PPAR-*α* ligands. Data generated using PPAR-*α* knockout mice indicate that this receptor regulates acute inflammation in vivo [27]. For example, PPAR-*α*-deficient mice have abnormally prolonged responses to different inflammatory stimuli [28]. Furthermore, fibrates have anti-inflammatory properties in vitro [29] and in vivo [30]. In particular, PPAR-*α* ligands can inhibit the expression of several proinflammatory genes such as IL-6, VCAM, and cyclooxygenase-2, in response to cytokine activation [30]. Moreover, the suppressive effect of PPAR-*α* ligands is mediated by inhibition of NF-*κ*B activation, in part by enhancing the expression of I*κ*B*α* [31]. It is important to note that synthetic and natural PPAR-*α* agonists can exert multiple biologic effects, including some which occur in a PPAR-*α*-independent fashion [32]. WY14643, like GW7647, shows excellent selectivity for murine and human PPAR-*α*.

Recent investigations have addressed the contribution of PPAR-*α* to the development of acute pleural and pulmonary inflammation and injury. We reported that when compared with wild-type mice, PPAR-*α* knockout mice experienced more severe pleural inflammation when subjected to intrapleural carrageenan administration. Specifically, the absence of a functional PPAR-*α* gene resulted in a significant augmentation of several inflammatory parameters (e.g., pleural exudate formation, mononuclear cell infiltration, and histological injury). Furthermore, PPAR-*α* ^−/−^ mice had enhanced the expression of tumor necrosis factor alpha (TNF-*α*), interleukin-1 beta (IL-1*β*), and FAS ligand in the pleural space post carrageenan administration [33].

Agonists for PPAR-*α* have been shown to reduce lipo-polysaccharide (LPS)- and cytokine-induced secretion of matrix metalloproteinase-9 (MMP-9) in human monocytes and rat mesangial cells, suggesting that this nuclear hormone receptor may play a beneficial role in controlling both tissue inflammation and remodeling. Consistent with this notion, Delayre-Orthez showed enhanced airway neutrophil and macrophage infiltration, elaboration of TNF-*α*, chemokines, and MMP 9 in PPAR-*α* ^−/−^ mice challenged with intranasal LPS, compared to that observed in similarly treated PPAR-*α* ^+/+^ mice. Conversely, pretreatment with the PPAR-*α* agonist fenofibrate reduced LPS-medicated airway inflammation, cytokine/chemokine expression and MMP-2 and -9 activity in bronchoalveolar lavage fluid [34]. Our laboratory has investigated the role of PPAR-*α* ligands in acute pulmonary inflammation using an experimental model of acute pancreatitis induced by cerulein. Intraperitoneal administration of cerulein in PPAR-*α* deficient mice resulted in severe infiltration of pancreatic and lung tissue with neutrophils (as measured by changes in myeloperoxidase activity), and enhanced expression of the adhesion molecules intercellular adhesion molecule-1 (ICAM-1), P-selectin, and growth factors TGF-*β* and VEGF in lung tissue, as compared to that observed in wild-type animals [35]. Interestingly, Jiang et al. have recently shown that acute lung injury in rats in response to LPS results in a reduced expression of PPAR-*α* mRNA and protein in the lung, raising the possibility that alterations in PPAR-*α* expression/activity may contribute to heightened inflammatory response [36].

Similar to effects in other models of pulmonary injury, PPAR-*α* appears to play a pivotal role in regulating the inflammatory response in experimental models of bleomycin-induced acute lung injury. Intratracheal administration of bleomycin in PPAR-*α* ^−/−^ mice resulted in a significant augmentation of TNF-*α*, IL-1*β*, and immunoreactive poly-ADP-ribose, as well as a loss of body weight and increased mortality. The dysregulated expression of poly-ADP-ribose is of particular relevance, as this molecule is synthesized from nicotinamide adenine dinucleotide (NAD) by poly-ADP ribose polymerase (PARP) during periods of oxidative stress, and enhanced PARP activity results in consumption of NAD^+^, ATP depletion, and ultimately cellular dysfunction. Conversely, the treatment of wild-type mice with WY14643 (1 mg/kg daily) prior to bleomycin administration significantly reduced the degree of lung injury, attenuated the rise in bleomycin-induced myeloperoxidase activity, and reduced the expression of TNF-*α*, IL-1*β*, and poly-ADP-ribose [37].

### 2.2. PPAR-*γ* and lung injury

In contrast to genetic models of PPAR-*α* deficiency, studies evaluating immunomodulatory effects of PPAR-*γ* have been limited by the absence of mice that are homozygous deficient for PPAR-*γ*, as these fetuses die in utero. For that reason, most studies assessing the role of PPAR-*γ* in inflammatory responses in vivo have relied on treatment with PPAR-*γ* agonists and/or antagonists or the use of mice that are heterozygous PPAR-*γ* deficient mice (PPAR-*γ* ^+/−^), which display reduced but not absence PPAR-*γ* activity.

As previously noted, the cyclopentenone prostaglandin 15d-PGJ_2_ functions as an endogenous ligand for PPAR-*γ*. We reported that 15d-PGJ_2_ (given at 10, 30, or 100 *μ*g/kg IP) in the carrageenan-induced pleurisy model exerted potent anti-inflammatory effects (e.g., inhibition of pleural exudate formation, mononuclear cell infiltration, delayed development of clinical indicators, and histological injury) in vivo. Furthermore, 15d-PGJ_2_ reduced the increase in nitrotyrosine and poly (ADP-ribose) polymerase and the expression of inducible nitric-oxide synthase and cyclooxygenase-2, as determined by immunohistochemistry, in the lungs of carrageenan-treated mice [38]. We also observed that rosiglitazone (given at 3, 10, or 30 mg/kg IP 15 minutes before carrageenan administration in the pleurisy model) exerted similar anti-inflammatory effects (e.g., inhibition of pleural exudate formation, mononuclear cell infiltration, and histological injury) in vivo as that observed with 15d-PGJ_2_. Furthermore, rosiglitazone reduced: (1) the increase in nitrotyrosine and poly (ADP-ribose) polymerase (PARP); (2) the expression of inducible nitric oxide synthase (iNOS), cyclooxygenase-2 (COX-2), intercellular adhesion molecules-1 (ICAM-1), and P-selectin in the lungs of carrageenan-treated rats. In order to elucidate whether the protective effect of rosiglitazone was causally related to activation of PPAR-*γ*, we investigated the effect of a PPAR-*γ* antagonist, bisphenol A diglycidyl ether (BADGE), on the protective effects of rosiglitazone. BADGE (30 mg/kg IP) administered 30 minutes prior to treatment with rosiglitazone significantly antagonized the suppressive properties of the PPAR-*γ* agonist [39].

In an animal model of severe haemorrhage and resuscitation, Abdelrahman et al. investigated the effects of 15d-PGJ_2_ administration on the development of multiple organ injury/dysfunction. Importantly, PPAR-*γ* agonist 15d-PGJ_2_ abolished the renal dysfunction and largely reduced the liver injury caused by hemorrhagic shock. In addition, 15d-PGJ_2_ attenuated lung and intestinal injury (as determined by histology) caused by haemorrhage and resuscitation [40].

We investigated the effects of rosiglitazone on the development of nonseptic shock caused by zymosan in mice. Treatment of mice with rosiglitazone (3 mg/kg IP, 1 and 6 hours after zymosan) attenuated the peritoneal exudation and the migration of polymorphonuclear cells caused by zymosan. Rosiglitazone also attenuated zymosan-induced lung dysfunction, as well as the increase in myeloperoxidase activity and malondialdehyde concentrations in the lung. To elucidate whether the protective effects of rosiglitazone occurred in a PPAR-*γ* specific fashion, we investigated the effect of a PPAR-gamma antagonist, GW9662, on the protective effects of rosiglitazone. GW9662 (1 mg/kg administered IP 30 minutes before treatment with rosiglitazone) significantly abolished the protective effect of rosiglitazone [41].

There exists convincing evidence that treatment with PPAR-*γ* agonists can also modulate pulmonary inflammation and tissue injury in response to systemic LPS administration and ischemia-reperfusion injury. For instance, experimental endotoxemia for 4 hours induced histological evidence of lung injury and edema formation, both of which were significantly attenuated by rosiglitazone pretreatment. The protective effects of rosiglitazone were correlated with the reduction by 71% and 84%, of the increase of myeloperoxidase and malondialdehyde, respectively, in the lung tissue. Furthermore, the pulmonary induction of nitric oxide was reduced by 82% of the increase related to lipopolysaccharide [42]. More recently, it has been shown that preischemic treatment with pioglitazone, a synthetic ligand of PPAR-*γ*, significantly attenuated ischemia/reperfusion (I/R)-induced lung injury in rats, including reductions in lung microvascular permeability, lipid peroxidation, tissue-associated polymorphonuclear leukocyte infiltration, and proinflammatory cytokine production. These findings can be explained, at least in part, by PPAR-*γ*-mediated inhibition of transcription factors such as NF-*κ*B [43], resulting in attenuated cytokine, chemokine and eicosanoid production, adhesion molecule expression, and as a consequence reduced inflammatory cell influx and injury to the alveolar capillary [44–47]. Another mechanism of protection afforded by the PPAR-*γ* agonist troglitazone in I/R lung injury is suppression of transcription factor early growth response gene-1 and its inflammatory gene targets such as interleukin-1*β*, monocyte chemotactic protein-1, and macrophage inflammatory protein-2 [48].

While the majority of studies have found potent anti-inflammatory properties of PPAR-*γ* agonists, observations made in several studies challenge this paradigm. Notably, Inoue et al. [49] demonstrated that pretreatment of mice with 15d-PGJ_2_ did not reduce pulmonary inflammation induced by intratracheal LPS administration. In fact, at the highest concentrations (1 mg/kg), 15d-PGJ_2_ paradoxicallyenhanced LPS-induced alveolar inflammation, pulmonary edema, and inflammatory cytokine expression. One possible explanation for the observed disparity in results may be attributable to PPAR-independent effects of selected agonists, dose-dependent toxicity or differences in the model systems used. The role of PPAR-*γ* in acute lung inflammation was also investigated in fluorescein isothiocyanate-treated mice. Here, pretreatment with pioglitazone (vehicle by oral gavage daily for 5 days) decreased the number of neutrophils recovered in bronchoalveolar lavage (BAL) by 50% 3 days after intratracheal challenge with fluorescein isothiocyanate. However, the decreased pulmonary inflammation was not associated withinhibition of the expression of inflammatory cytokines (TNF-*α*, macrophage inflammatory protein-2, *KC*, IL-12, or IFN-*γ*) in either BAL fluidor whole lung homogenates [50]. The authors speculated that the possible mechanism by which a PPAR-*γ* ligand suppresses inflammation in the absence of changes in cytokine expression was by a direct effect on migration of neutrophils (and possibly other leukocytes) in response to endogenous chemoattractants [50]. In the FITC model, treatment with pioglitazone also had only modest suppressive effects on alveolar-capillary leak or subsequent fibroproliferation. The disparate effects of PPAR-*γ* agonists on inflammation relative to alveolar capillary injury and repair may be attributable to direct effects of PPAR-*γ* activation on alveolar epithelial cells. Treatment of A549 alveolar type II-like epithelial cells with 15d-PGJ_2_ or TZDs, or forced expression of a constitutively active PPAR-*γ* has been shown to suppress NF-*κ*B transcriptional activity and decreased inflammatory cytokine and chemokine production. However, incubation of these cells with PPAR-*γ* ligands also suppressed alveolar epithelial cell proliferative responses. Collectively these data suggest that beneficial anti-inflammatory properties of PPAR-*γ* in ALI may be partially offset by growth inhibitory effects on alveolar epithelial cells, responses that are necessary for repair of an injured alveolar-capillary membrane.

Orderly lung remodeling is required for restoration of an intact alveolar-capillary membrane after injury. Fibroblasts are one of the key effector cells in this process. However, the differentiation of fibroblasts to myofibroblasts can result in excessive and uncontrolled production of collagen and other extracellular matrix components, leading to fibrosis. Importantly, PPAR-*γ* agonists have been shown to block two of the most important profibrotic activities of TGF-*β* on pulmonary fibroblasts; myofibroblast differentiation and production of excess collagen. Both natural (15d-PGJ_2_) and synthetic (ciglitazone and rosiglitazone) PPAR-*γ* agonists inhibited TGF-*β*-driven myofibroblast differentiation in human lung fibroblasts, as determined by alpha-smooth muscle actin expression. PPAR-*γ* agonists also potently attenuated TGF-*β*-induced type I collagen protein production [51]. Transfection with a dominant-negative PPAR-*γ* construct partially reversed the inhibition of myofibroblast differentiation by 15d-PGJ2 and rosiglitazone, but the irreversible PPAR-*γ* antagonist GW-9662 did not, suggesting that the antifibrotic effects of the PPAR-*γ* agonists are mediated through both PPAR-*γ*-dependent and independent mechanisms.

Observations made in several studies suggest that the activation of PPAR-*γ* may exert both anti-inflammatory and antifibrotic effects in vivo. Mice subjected to intratracheal administration of bleomycin develop marked lung injury followed by fibrosis. An increase in immunoreactivity to nitrotyrosine, poly (ADP ribose) polymerase (PARP), and inducible nitric oxide synthase as well as a significant loss of body weight and mortality was observed in the lung of bleomycin-treated mice. Administration of the two PPAR-gamma agonists rosiglitazone (10 mg/kg IP) or 15d-PGJ_2_ (30 *μ*g/kg IP) significantly reduced: (1) the loss of body weight; (2) mortality rate; (3) infiltration of the lung with polymorphonuclear neutrophils (myeloperoxidase activity); (4) edema formation; (5) histological evidence of lung injury and fibroproliferation; and (6) nitrotyrosine, PARP, and inducible nitric oxide synthase formation [52]. Pretreatment with the PPAR-gamma competitive antagonist BADGE substantially mitigated the effect of the two PPAR-gamma agonists, indicating a PPAR-*γ* specific response. Our findings are in agreement with Ando et al. [53], who demonstrated that the intravenous injection of prostaglandin D synthase (PGDS) cDNA-expressing fibroblasts significantly reduced lung edema, BAL leukocytes, and pulmonary collagen 4 weeks after intratracheal instillation of bleomycin. Moreover, this attenuated lung response to bleomycin was quite similar to that seen in animals pretreated with 15d-PGJ_2_, the nonenzymatic metabolite of PGD_2_, suggesting that these naturally occurring ligands exert relevant effects on the fibroproliferative response in vivo.

## 3. CONCLUSION

The subsequent tissue response to acute and chronic lung injury involves an intricate series of events including immune cell infiltration, release of injurious host-derived molecules such as reactive oxygen and nitrogen species, and high permeability edema formation. In addition, fibroproliferative repair is characterized by myofibroblast transdifferentiation and the deposition of extracellular matrix proteins. Failure to initiate, maintain, or stop this repair program has dramatic consequences such as cell death or exuberant wound repair. PPARs appear to be critical regulators of host inflammatory and reparative responses, and these transcriptional factors may be activated by lipid mediators produced in response to lung injury. The generation of better transgenic model systems, including conditional and site-specific transgenic mouse models, are required to more precisely define the contribution of PPAR-*γ* and other PPAR family members to disease pathogenesis in ALI and other inflammatory lung diseases. This class of nuclear hormone receptors may serve as important targets for therapeutic intervention in the treatment of patient with both acute and chronic inflammatory disorders of the lung.
